# The burden of high workload on the health-related quality of life among home care workers in Northern Sweden

**DOI:** 10.1007/s00420-020-01530-9

**Published:** 2020-03-05

**Authors:** André Sjöberg, Anita Pettersson-Strömbäck, Klas-Göran Sahlén, Lars Lindholm, Fredrik Norström

**Affiliations:** 1 Department of Epidemiology and Global Health, Umeå University, SE-901 87, Umeå, Sweden; 2 Department of Psychology, Umeå University, Umeå, Sweden

**Keywords:** Quality-adjusted life years, Demand–control–support model, Psychosocial factors, Propensity scores

## Abstract

**Objective:**

Previous studies have shown that high workload affects health negatively. However, studies are lacking among home care workers. The aim of this study is to examine the burden of perceived workload on health-related quality of life (HRQoL) among home care workers and to determine whether psychosocial factors modify such a relationship.

**Methods:**

A cross-sectional study was conducted in which 1162 (58% response rate) home care workers participated. The psychosocial factors were measured by QPSnordic. HRQoL was measured by EuroQol 5 dimensions, from which responses were translated into quality-adjusted life year scores (QALY). Propensity scores were used with absolute risk differences (RD). Stratified analysis was used to test the buffer hypothesis of the demand–control–support model.

**Results:**

Personnel with a high workload had a statistically significant 0.035 lower QALY than personnel with a normal workload. This difference was also statistically significant for the Visual Analogue Scale (RD 5.0) and the mobility (RD 0.033) and anxiety/depression scales (RD 0.20) dimensions of EQ-5D. For QALY, the effect of a high workload compared to a normal workload was higher, with low (RD 0.045, significant) compared with high (RD 0.015, non-significant) social support; while it was similar, and non-significant results, for low and high control.

**Conclusions:**

Our study shows that lowered work burden would be beneficial for home care personnel. Furthermore, our results suggest that interventions aimed at increasing social support could reduce work-related illness.

## Introduction

Sweden is currently facing a demographic transition with an increasing elderly population and, thus, an increase in life expectancy. As a consequence, the total number of elderly people receiving assistance from home care has also been growing (The Swedish National Board of Health and Welfare 2018). This has put many municipalities’ home care systems under a lot of stress. The workload, both physical and psychosocial, has increased significantly and there have been several reports about poor working environment (Skovdahl et al. 2008), employee burnout, high rates of sick leave, stress of conscience and high workload (Gustafsson et al. 2010; Szebehely et al. 2017; Trydegård 2012). For example, Szebehely with colleagues has shown that the number of patients a home care worker must meet during one workday has increased steadily, from around four patients in the 1980s, to six–seven in the early 2000s to today, where a home care worker in average must assist almost 13 patients per day (Szebehely et al. 2017).

In Sweden, it is the municipalities that are responsible for help and support in daily life for old people. County councils and municipalities share the responsibility for medical care in old age. The types of interventions available, organisation of caregivers and the extent of the interventions may differ between municipalities, but all the municipalities are obligated by the Social Service Act (SFS 2001:453) to offer help and support in daily life to the ones who are unable to care for themselves. The home care is provided through a publicly regulated and tax-funded system, but the actual care can be provided by a mix of public, non-profit and for-profit organisations (Winblad et al. 2017). Not only the number of elderly people receiving home care has increased, but also they live in their home for a longer time and receive more advanced health care in their homes (The Swedish National Board of Health and Welfare 2018). For this reason, both the workload and work complexity have increased for home care workers. Elderly care is one of the largest sectors in Sweden, and among the employees, approximately 250,000 health care workers are estimated to be working in it (Suvanto et al. 2017). Those working in home care are mainly assistant nurses with a high school health education or assistant health workers without a formal college or university education adapted to the work. Health and medical interventions are done by licensed nurses or by the home care workers with delegation by a nurse.

The previous research on workload is extensive and high workload has been shown to be negatively associated with multiple health measurements, such as job satisfaction, mental well-being, job strain, depression, distress, fatigue, emotional exhaustion and physical symptoms (Bowling et al. 2015; SBU 2014, 2015). In our study, workload is defined in broad terms and include both quantitative and qualitative dimensions (Cooper et al. 2001). The quantitative demands are related to the amount of work and the qualitative demands to the difficulty of the work (Bowling et al. 2015).

Despite reports about how high workload affects health among Swedish health care personnel, there is a lack of studies done within home care and also overall for health-related quality of life (HRQoL). HRQoL assesses how individuals’ general well-being is affected by a certain exposure and includes both perceived physical- and mental health (CDC 2000). A connection has been shown between poor HRQoL and high workload among nurses in both Greece and China; while, this has not previously been studied within Swedish health care (Sarafis et al. 2016).

Several occupational psychosocial factors, such as workload, social support, role ambiguity, job control and work-life conflicts have been shown to be related to both psychological and physical health outcomes (Alarcon 2011; Bernal et al. 2015; Sverke et al. 2016). Even with the extensive research in the field, further research is needed on the correlates and consequences of workload (Bowling et al. 2015).

The job demand–control–support model (JDC) is an extension on Karasek’s demand–control model (DCS), which is one of the most influential theories about occupational well-being (Karasek 1979). The job demand–control model claims that occupational well-being is based on the interaction between two factors, job demand and job control. Johnson and Hall then added social support to the model, which created the demand–control–support model (Johnson and Hall 1988). Both of these models are based on the same two hypotheses, the strain and the buffer hypothesis. The strain hypothesis claims that the most negative outcomes will be found in employees who are exposed to high job demands, low job control and low social support. In the demand–control–support model, this type of setting is labelled as isolation strain (Or ISO strain). The buffer hypothesis claims that control and social support will have a “buffering” or moderating effect on the relationship between demand and the health outcome (Van der Doef and Maes 1999).

According to Szebehely, at the same time, as the workload in home care for old people has been increasing, the workers’ ability to control their daily work has also been decreasing (Szebehely et al. 2017). This would, according to the JDC model, have a major negative impact on their health. But, how or to what degree control and social support moderates the well-being of home care workers has not yet been tested in this setting.

In previous studies performed in the Nordic countries, Sweden has been found to have the lowest proportion of full-time employments and has the highest use of split shifts (Szebehely et al. 2017). Managers in home care have increasing difficulties in recruiting and keeping educated staff and, according to a report from the municipal workers’ union Kommunal, their members describe a work environment characterised by high stress, understaffing and lack of leadership (Kommunal 2015). One third of the respondents who where under 35 years old reported that they did not want to continue to work in the home care sector in three years’ time. Furthermore, to recruit personnel, the Swedish government states that a good and secure work environment is necessary (The Ministry of Health and Social Affairs Sweden 2018).

With this large number of reports on the poor working environment from the Swedish home care sector and increasing difficulties in employing trained staff, it is crucial to examine how workload and psychosocial factors affect home care workers’ health. Much of the previous research on the DCS model has been on a large-scale representative research population, something that has been described as a weakness because of the large diversity in individual occupations in the field studied (deJonge and Kompier 1997).

The main aim of this study is to examine the burden of perceived workload on HRQoL among home care workers in Northern Sweden. The secondary objective is to determine whether, and to what extent, the factors in the JDC model modify the association between workload and HRQoL. This study will contribute knowledge about the current situation among health workers in Swedish home care when it comes to health, workload and well-being, and it will also strive to further the current research on occupational health by studying the health impact of psychosocial factors in a new setting.

## Methods

### Sample and data collection

In our study, all municipalities from three counties in the northern part of Sweden, namely Västerbotten, Jämtland and Västernorrland, were invited to participate, and 16 of the municipalities were part of the current study. In participating municipalities, all employees within their municipal home care were invited to participate in a cross-sectional questionnaire study. The questionnaire contained questions related to demographics, work environment, daily work activity and health-related quality of life. Questionnaires were distributed in October 2017 to the home care organisation within participating municipalities. In the municipalities, most of the staff were given a questionnaire at their workplace, usually to be filled in during workplace meetings, but also to a lesser extent questionnaires were posted to their homes. Questionnaires were distributed to around 2000 home care workers (the exact numbers are not available as not all home care organisations were able to document exact numbers to whom they distributed the questionnaire), with 1154 responding before the end of January 2018 (response rate 58%). We used questions belonging to the General Nordic Questionnaire for Psychological and Social Factors at Work (QPSnordic) to define workload, social support and work control, and the EuroQol 5 dimensions (EQ-5D) instrument for HRQoL, gender, age, health education, marital status, and tenure in the occupation.

### Psychosocial measures

Psychosocial factors were measured by items included in the General Nordic Questionnaire for Psychological and Social Factors at Work (QPSnordic), which is a “…general questionnaire for measuring psychological and social factors at work, including job and organisation characteristics, as well as individual work-related attitudes.” (Dallner et al. 2000). The full version of QPSnordic contains 129 items, where 11 items are background questions, 38 items are individual questions and 80 questions are distributed in 13 scales related to psychosocial and social factors at work. In our questionnaire, we included 21 of the latter 80 questions, which all had five response alternatives: “Very seldom or never”, “Rather seldom”, “Sometimes”, “Rather often”, and “Very often or always”.

In the current study, 13 questions from QPSnordic were used, distributed in three scales that measure the psychosocial factors of workload, social support and control (Dallner et al. 2000). We defined Workload based on four questions belonging to the two subscales Quantitative-, and Learning demands of the QPSnordic scale Job demands. Control is defined based on items from the two subscales: Control of Decision and Control of Work Speed. Social support is based on two subscales: Support from Superior and Support from co-workers. In Table 1, questions in the different subscales are presented. In the current study, we settle for analysing data at an aggregated level and are not exploring differences at a question level other than descriptively.

**Table 1:** Scales, subscales and items in a reduced QPSnordic version used in current study

Workload—Quantitative demands
Is your workload so unevenly distributed that work is getting bogged down?
Do you have too much to do?
Workload—Learning demands
Are your work tasks too difficult for you?
Perform tasks for which you would need more education
Control—Decision
Can you influence the amount of work you get?
Can you influence decisions that are important to your work?
Control—Work speed
Can you decide your work rate yourself?
Can you decide when to take a break?
Social support—Support from superior
If you need, do you receive support and assistance with your work from your immediate boss?
Does your immediate boss appreciate your work performance?
Social support—Support from co-workers
If you need it, do you receive support and assistance with your work from co-workers?

All questions had five response alternatives: very seldom or never, rather seldom, sometimes, rather often, and very often or always

### Health-related quality of life measure

HRQoL was assessed by EQ-5D, which is a validated generic non-disease-specific instrument (Devlin and Brooks 2017), which includes both a descriptive system and a visual analogue scale (EQ-VAS). The descriptive system consists of five questions, which corresponds to the five dimensions of health: mobility, self‐care, usual activities, pain/discomfort and anxiety/depression, with each dimension valued by a three-point severity scale ranging from no problems to severe problems. An EQ-5D index score, which correspond to a quality-adjusted life year score, can be calculated using a country tariff with two anchor points, 0 (death) and 1 (full health), and in this study, we used tariffs from the United Kingdom and refer to the score as QALY (Dolan 1997).

### Covariates

Woman was used as exposure for gender. There were four possible responses to health education for the participants. In our analyses, the outcomes “assistant nurse” and “other health education” were defined as “assistant nurse”, and the outcomes “nursing assistant” and “no health education” were defined as “other education” and used as exposure. In Sweden, an assistant nurse has an education corresponding to at least 2 years of upper secondary diploma in health and social care, while nursing assistants have a more limited, often workplace-related, training. Marital status was classified as either living single, used as exposure, or with a partner, where single consisted of both those who indicated “single” and “single with children”. Tenure in the home care evoked responses of “less than 1 year”, “1–5 years”, “6–15 years” and “more than 15 years” of experience. Based on age and tenure, a variable was created where at most 5 years’ tenure in home care (regardless of age) was used as exposure and more than 5 years’ tenure was divided into three groups, based on age, namely at most 35 years of age, 36–54 years of age and at least 55 years of age.

### Statistics

We defined individuals to have a high workload as the median of the four workload questions being at least “sometimes”, and treated it as exposed in analyses, i.e., a third alternative or more of the five listed, else individuals were defined as having a normal workload. In sensitivity analyses, we varied the definition of workload with the questions’ ordinal responses coded as 1–5. In the first alternative, high workload was defined as an average value above 3, and in the second alternative, it was defined as an average value above 2.5.

We defined high control as having a mean value on the ordinal scales for the control variables of 3 or above, unless individuals were defined as having low control. We defined high social support as having a mean value on the ordinal scales for the social support variables of more than 3, unless individuals were defined as having low social support.

In our analyses, our main health outcome was QALY and EQ-VAS, but we also presented results for the EQ-5D dimensions themselves. In all analyses, we required valid responses to the outcome (health), the main exposure (workload) and covariates (gender, health education, marital status, and tenure in the home care). We, therefore, included 1029 (89% of the responses) of 1154 responders for analyses of the descriptive system, including QALY, and 1002 (87% of the responses) of 1154 responders for EQ-VAS analyses. The number of missing or non-valid responses was for the respective variables: 44 for any of the EQ-5D questions, 44 for any of the questions used to define workload, 7 for sex, 24 for education, 11 for marital status, 6 for tenure and 19 for birth year (used to derive age). Requiring responses for covariates reduced the sample size with 45 participants (only 4.2% of participants with valid responses to EQ-5D and workload questions). For sub-analyses where social support or control was used, the number of responses was further reduced due to participants not responding to one or more of these questions.

We dichotomized answers to the questions to “no problems”, and “problems” where the latter alternative included responses corresponding to moderate and severe problems. Analyses were not performed for the EQ-5D dimension self-care, as such a limitation is unlikely in the occupation, which was also backed up by questionnaire responses, as only five participants responded moderate or severe problems with self-care.

We used a propensity score weighting to estimate the effect of workload on HRQoL (Lunceford and Davidian 2004). A more detailed description of our use of propensity scores is available elsewhere (Norström et al. 2017). To derive the propensity scores, which correspond to the probability of being exposed, we used our covariates in a logistic regression. In our case, being exposed corresponds to having a high workload. Using propensity scores in our study results in a quasi-experimental approach. Additionally, we present results with logistic regression, adjusting for our covariates, with 95% confidence intervals, for the descriptive questions of the EQ-5D instrument.

We used an inverse probability weight (IPW) estimator to estimate the risk difference using counterfactual arguments, i.e., the effect on health if individuals move from normal to high workload, as suggested by Lunceford and Davidian (Lunceford and Davidian 2004):$RD_{IPW} = \left( {\sum\limits_{i = 1}^{n} {\frac{{X_{i} }}{{PS_{i} }}} } \right)^{ - 1} \sum\limits_{i = 1}^{n} {\frac{{Y_{i} X_{i} }}{{PS_{i} }}} - \left( {\sum\limits_{i = 1}^{n} {\frac{{1 - X_{i} }}{{1 - PS_{i} }}} } \right)^{ - 1} \sum\limits_{i = 1}^{n} {_{i} \frac{{Y(1 - X_{i} )}}{{1 - PS_{i} }}}$, where *Y* refers to the outcome (HRQoL), *X* to the exposure (workload), PS to the propensity scores, and RD to the risk difference, which estimates the absolute difference between groups. The standardised difference was calculated, both with and without a weight, to assess the balance of covariates between the groups of high and low workload for each covariate (Austin and Stuart 2015; Norström et al. 2017). A standardised difference below 10% is according to Austin and Stuart considered by some experts to be a negligible imbalance.

Descriptive statistics were used to present the characteristics of the sample. Analyses were performed also at group level for not only our covariates but also stratified for control and social support, the latter two stratifications to evaluate the moderating effect of control and support on the association between workload and HRQoL. Pearson’s *χ*^2^-test was used to test whether the exposure variable (workload) was associated with covariates. Student’s *t*-test was used to test differences in age with respect to QALYs between those with high and normal workloads. Interactions between variables were not considered in any of our analyses. We did not experience problems due to collinearity between variables, and hence, all candidate variables were kept in the analyses.

R Studio was used for statistical analyses (R Studio, Boston, MA), with its GLM procedure used for logistic regression, where confidence intervals were derived with the profile likelihood (R Core Team 2015). The Bootstrap technique with replacement was used to derive the mean square error, confidence intervals, which corresponded to the 2.5% and 97.5% percentiles, and *p*-values from 10,000 replicates (Davison and Hinckley 1997). Statistical significance was defined at the 5% level.

## Results

### Characteristics of the study population

Those considered to have a high workload had a statistically significantly lower age than those considered to have a normal workload (41 years vs. 44 years). Among participants, there were more women (84%) than men (16%), and a majority (71%) were assistant nurses (Table 2). Those with low control in their work had a statistically significantly higher proportion of individuals with a high workload than those with high control. Also for those with a low social support, there was a statistically significantly higher proportion of individuals with a high workload compared with participants who had a high social support.

**Table 2:** Characteristics of the study population (*n* = 1029)

	High workload (*n* = 291)			Normal workload (*n* = 738)			*p*
	*n*		%	*n*		%	
Gender							
Man (*n* = 163)	51		31	112		69	0.353
Woman (*n* = 866)	240		28	626		72	
Marital status							
Married (*n* = 699)	187		27	512		73	0.113
Single (*n* = 330)	104		32	226		68	
Tenure							
< 1 year (*n* = 85)	20		24	65		76	0.067
1–5 years (*n* = 297)	101		34	196		66	
6–15 years (*n* = 327)	88		27	239		73	
> 15 years (*n* = 320)	82		26	238		74	
Health education							
Assistant nurse (*n* = 735)	191		26	544		74	0.010
Other education (*n* = 294)	100		34	194		66	
Employment form							
Permanent (*n* = 868)	246		28	622		72	0.933
Temporary (*n* = 74)	22		30	52		70	
By the hour (*n* = 85)	23		27	62		73	
Tenure and age							
Up to 5 years of experience (*n* = 382)	121		32	261		68	0.051
More than 5 years of experience and ≤ 35 years of age (*n* = 117)	32		27	85		73	
More than 5 years of experience and 36–54 years of age (*n* = 309)	91		29	218		71	
More than 5 years of experience and ≥ 55 years of age (*n* = 221)	47		21	174		79	
Control							
Low (*n* = 550)	193		35	357		65	< 0.001
High (*n* = 457)	92		20	365		80	
Social support							
Low (*n* = 320)	139		43	181		57	< 0.001
High (*n* = 697)	151		22	546		78	
	Mean	Median	SD	Mean	Median	SD	
Age*	41	42	14	44	47	14	

Control and social support are defined based on questionnaire responses. Due to missing data, eligible responses are less than 1029. *p*-value using *χ*-test

*SD* standard deviation

*Significance at 5% using *t*-test

### Workload

In our study, after removal of participants who did not have valid responses to all questions used in our analyses, 291 (28%) participants were considered to have a high workload (Table 2). The distribution of the responses to the workload questions varied, with few experiencing problems with work tasks that were too difficult (1.1% at least rather often experiencing problems) and considering their work tasks to require more training (7.1% at least rather often experiencing problems); while, unevenly distributed workload (20% at least rather often experiencing problems) and too much to do (25% at least rather often experiencing problems) were more of a problem for the home care workers (Appendix). Thus, there were more problems reported related to the quantitative than the learning demands in our study. The problems experienced for the four different workload-related issues varied to some extent between groups of individuals in the variation seeming to be mainly related to gender, tenure and health education.

### Health-related quality of life

For the responses to the five dimensions of the EQ-5Ds descriptive system, there were few who expressed extreme problems among either those with a high or a normal workload; while for both pain/discomfort and anxiety/depression, it was common to find some problems (Table 3). For the mobility and usual activities, there were not more than 8% who at least experienced some problems; while for logical reasons, there were almost none (five individuals) who expressed problems with self-care. When comparing those with high and normal workloads, there was a numerically large difference for anxiety/depression (43% among those with a high workload, and 22% among those with a normal workload expressed problems) and usual activities (8% among those with high workload and 4% among those with a normal workload expressed problems) dimensions. The mean QALY and EQ-VAS were both higher for those with a normal workload than a high workload (Table 3).

**Table 3:** Responses to EuroQol 5 dimensions (*n* = 1029)

	High workload (*n* = 291)			Normal workload (*n* = 738)		
	*n*		%	*n*		%
Mobility						
No problems (*n* = 966)	269		92.4	697		94.4
Some problems (*n* = 62)	21		7.2	41		5.6
Extreme problems (*n* = 1)	1		0.3	0		-
Self-care						
No problems (*n* = 1024)	289		99.3	735		99.6
Some problems (*n* = 2)	1		0.3	1		0.1
Extreme problems (*n* = 3)	1		0.3	2		0.3
Usual activities						
No problems (*n* = 977)	268		92.1	709		96.0
Some problems (*n* = 51)	23		7.9	28		3.8
Extreme problems (*n* = 1)	0		-	1		0.1
Pain/discomfort						
No problems (*n* = 427)	118		41.5	309		41.9
Some problems (*n* = 571)	165		56.7	406		55.0
Extreme problems (*n* = 31)	8		2.7	23		3.1
Anxiety/depression						
No problems (*n* = 738)	166		57.0	572		77.5
Some problems (*n* = 279)	117		40.2	162		22.0
Extreme problems (*n* = 12)	8		2.7	4		0.5
	Mean	Median	SD^b^	Mean	Median	SD^b^
QALY score^a^	0.799	0.796	0.19	0.833	0.796	0.17
EQ-VAS^c^ (*n* = 1002)	75.7	80	24.9	80.7	85	15.5

^a^Quality-adjusted life years score

^b^Standard deviation

^c^For EuroQol Visual Analogue Scale (EQ-VAS), 280 with a high work demand and 722 with a normal work demand were included

### Workload and health-related quality of life

There was a statistically significantly lower QALY if having a high workload compared with a normal workload (Table 4), which in health-economic terminology corresponds to a loss of 0.35 quality-adjusted life years during a 10-year period. Also for EQ-VAS, there was a statistically significant poorer health reported from those with a high workload than a normal one, and there was even a larger absolute difference than it was for QALY (absolute difference of 5.0% for EQ-VAS compared to 3.5% for QALY). For the dimensions of EQ-5D (Table 4 and Appendix), poorer health was reported for those with a higher workload with a statistical significance for usual activities (an absolute increase in 3.3% with problems and an odds ratio of 2.0 if individuals went from normal to high workload) and for anxiety/depression (an absolute increase in 20% with problems and an odds ratio of 2.6 if individuals went from normal to high workload). For the mobility and pain/discomfort dimensions, there was no statistical significance, with results indicating at most a marginal negative effect from a high workload for personnel on these dimensions.

**Table 4:** Effect of health-related quality of life from a high workload within homecare (*n* = 1029^a^)

Health measure	Risk difference^b^	Confidence interval	*p*	Mean squared error
Quality-adjusted life years score	− 0.035^c^	[− 0.060 to − 0.011]	0.005	0.00015
EQ-5D^d^—Mobility	0.025^e^	[− 0.010–0.062]	0.177	0.00034
EQ-5D^d^—Usual activities	0.033^e^	[0.001–0.065]	0.043	0.00026
EQ-5D^d^—Pain/discomfort	0.024^e^	[− 0.042–0.090]	0.462	0.00112
EQ-5D^d^—Anxiety/depression	0.203^e^	[0.139–0.272]	< 0.001	0.00114
EQ-VAS^f^	− 5.03^c^	[− 7.84 to − 1.73]	0.007	2.459

^a^Of whom, 291 are defined with a high work load and 738 with a normal work load

^b^Propensity scores were derived using gender, education level, marital status, and a variable combining age and tenure in occupation

^c^A risk difference below 0 means more problems with health-related quality of life when having a high workload than having a normal workload

^d^EuroQol 5 dimensions. Responses dichotomized to no problems and at least moderate problems. Problems with each of the dimensions were: 63 for mobility, 52 for usual activities, 602 for pain/discomfort and 291 for anxiety/depression

^e^A risk difference above 0 means more problems with health-related quality of life when having a high workload than having a normal workload

^f^EuroQol Visual Analogue Scale, *n* = 1002 responses of whom 280 are defined with a high work load and 722 with a normal work load

In our sensitivity analyses, the first alternative definition (the mean value above 3 for the workload questions), where fewer participants (*n* = 105) were classified as having a high workload, showed a statistically significant negative effect from high workload with a larger negative effect of 0.067 for QALY and 8.0 for EQ-VAS than for our main definition (Appendix). There was also a comparably higher negative effect for the EQ-5D dimensions than for the estimated effects for our main definition. For the second alternative definition (*n* = 293 participants with high workload based on a mean value above 2.5 for the workload questions), the negative effect for those with high workload compared with a normal workload was generally also larger than for the main definition, but not when compared with the first alternative workload definition for all estimates but for usual activities.

The balance of the covariates was improved with the propensity scores. The standardised difference ranged from 1.6 to 21% when weights for the QALY score estimates were not applied and from 0.02 to 12% when such weights were applied (Appendix). The balance was not optimal for “at most one year’s tenure”. For all variables that were used for the derivation of propensity score weights, the imbalance was very low (below 1%).

### Workload and health-related quality of life at group level

For QALY, EQ-VAS and for the anxiety/depression dimension, there was a statistically significant negative effect of high workload for women; while, the effect of high workload was both smaller and non-significant for these measures for men (Tables 5, 6, 7). For marital status, there was a similar effect for married and single in terms of QALY, but it was only statistically significant for single participants; while for EQ-VAS, it was only statistically significantly poorer health for single participants due to a high workload. For health education, there was in general a larger negative effect from high workload for those who were not assistant nurses compared to the assistant nurses. This was most notably for EQ-VAS, where there was a statistically significant negative effect of 7.7% poorer health if having a high workload compared to a normal workload for those who were not assistant nurses; while, there was only a marginal non-significant difference for assistant nurses. For tenure and age, the effect of a high workload was largest for those who were older and had at least 5 years of experience within home care for both QALY and EQ-VAS. For the pain/discomfort dimension of EQ-5D, there were no statistical significances for the stratified results; while it was only for those with more than 5 years of experience and short time in the home care occupation that statistically significant negative effects of high workload could not be observed for the anxiety/depression dimension (Table 6).

### Health-related quality of life in relation to the job demand–control–support

Individuals experiencing low social support seems to be more affected by high workload for QALY than those experiencing high social support, as there was only a statistical significance for low and not high social support, with an effect estimate of 0.045 compared to 0.015 (Table 5). The same pattern existed also for anxiety/depression, where a high workload increased the absolute numbers of individuals with problems who had low social support with 26% compared to 13% for those with high social support, both increases being statistically significant (Table 6). Also, for EQ-VAS, a similar distinction between low and high social support seemed to exist (Table 7). For analyses based on level of control in the personnel’s working situation, results were similar apart from EQ-VAS, where low control showed a statistically significant effect, while high control did not, though, in effect the size difference between the estimates was small (Tables 5, 6, 7).

**Table 5:** Stratified results of the effect of high workload for home care personnel on health on quality-adjusted life-year scores (QALY) (*n* = 1029)

Stratification group	Risk difference^a^	Confidence interval	*p*
Gender			
Man (*n* = 163)	− 0.005	[− 0.069, 0.054]	0.877
Woman (*n* = 866)	− 0.042	[− 0.070, − 0.016]	< 0.001
Marital status			
Married (*n* = 699)	− 0.035	[− 0.079, 0.007]	0.113
Single (*n* = 330)	− 0.035	[− 0.064, − 0.007]	0.016
Health education			
Assistant nurse (*n* = 735)	− 0.031	[− 0.060, − 0.003]	0.033
Other education (*n* = 294)	− 0.046	[− 0.093, − 0.003]	0.038
Tenure and age			
Up to five years of experience (*n* = 382)	− 0.016	[− 0.052, 0.020]	0.387
More than 5 years of experience and ≤ 35 years of age (*n* = 117)	− 0.010	[− 0.085, 0.062]	0.815
More than 5 years of experience and 36–54 years of age (*n* = 309)	− 0.060	[− 0.110, − 0.016]	0.008
More than 5 years of experience and ≥ 55 years of age (*n* = 221)	− 0.047	[− 0.096, 0.004]	0.073
Control^b^			
Low (*n* = 550)	− 0.024	[− 0.058, 0.008]	0.134
High (*n* = 457)	− 0.034	[− 0.072, 0.002]	0.062
Social support^c^			
Low (*n* = 320)	− 0.045	[− 0.090, − 0.0004]	0.048
High (*n* = 697)	− 0.015	[− 0.044, 0.013]	0.300

^a^A risk difference below 0 means more problems with QALY when having a high workload than having a normal workload

^b^There were *n* = 1007 of the included individuals who responded to the control questions

^c^There were *n* = 1017 of the included individuals who responded to the social support questions

**Table 6:** Stratified results for the effect of high workload on health for the EQ-5D dimensions (*n* = 1029)

Stratification group	Risk difference^a^	*p*
Pain/discomfort		
Gender		
Man (*n* = 163)	0.045	0.60
Woman (*n* = 866)	0.024	0.51
Marital status		
Married (*n* = 699)	0.027	0.66
Single (*n* = 330)	0.030	0.46
Health education		
Assistant nurse (*n* = 735)	0.019	0.62
Other education (*n* = 294)	0.034	0.57
Tenure and age		
Up to 5 years of experience (*n* = 382)	− 0.032	0.56
More than 5 years of experience and ≤ 35 years of age (*n* = 117)	0.008	0.95
More than 5 years of experience and 36–54 years of age (n = 309)	0.074	0.18
More than 5 years of experience and ≥ 55 years of age (*n* = 221)	0.084	0.28
Control^b^		
Low (*n* = 550)	− 0.016	0.72
High (*n* = 457)	0.066	0.22
Social support^c^		
Low (*n* = 320)	0.009	0.86
High (*n* = 697)	0.013	0.78
Anxiety/depression		
Gender		
Man (*n* = 163)	0.072	0.32
Woman (*n* = 866)	0.229	< 0.01
Marital status		
Married (*n* = 699)	0.159	< 0.01
Single (*n* = 330)	0.236	< 0.01
Health education		
Assistant nurse (*n* = 735)	0.197	< 0.01
Other education (*n* = 294)	0.216	< 0.01
Tenure and age		
Up to 5 years of experience (*n* = 382)	0.140	< 0.01
More than 5 years of experience and ≤ 35 years of age (*n* = 117)	0.067	0.53
More than 5 years of experience and 36–54 years of age (*n *= 309)	0.279	< 0.01
More than 5 years of experience and ≥ 55 years of age (*n* = 221)	0.306	< 0.01
Control^b^		
Low (*n* = 550)	0.184	< 0.01
High (*n* = 457)	0.196	< 0.01
Social support^c^		
Low (*n* = 320)	0.257	< 0.01
High (*n* = 697)	0.132	< 0.01

^a^The risk difference presents the increase in the proportion of individuals with health problems due to a high workload

^b^There were *n* = 1007 of the individuals included who responded to the control questions

^c^There were *n* = 1017 of the included individuals who responded to the social support questions

**Table 7:** Stratified results for the effect of high workload on health on EuroQol 5D Visual Analogue Scale (*n* = 1002)

Stratification group	Risk difference^a^	Confidence interval	*p*
Gender			
Man (*n* = 158)	2.49	[− 9.89, 23.0]	0.864
Woman (*n* = 844)	− 6.15	[− 8.68, − 3.43]	< 0.001
Marital status			
Married (*n* = 679)	− 0.76	[− 8.24, 9.99]	0.801
Single (*n* = 323)	− 6.94	[− 9.81, − 3.98]	< 0.001
Health education			
Assistant nurse (*n* = 716)	− 3.81	[− 7.52, 0.78]	0.096
Other education (*n *= 286)	− 7.70	[− 11.8, − 3.49]	< 0.001
Tenure and age			
Up to 5 years of experience (*n* = 369)	− 2.81	[− 8.72, 4.95]	0.412
More than 5 years of experience and ≤ 35 years of age (*n* = 117)	− 2.97	[− 9.89, 4.03]	0.390
More than 5 years of experience and 36–54 years of age (*n* = 304)	− 7.82	[− 11.8, − 3.46]	< 0.001
More than 5 years of experience and ≥ 55 years of age (*n* = 212)	− 6.22	[− 11.7, − 1.11]	0.018
Control^b^			
Low (*n* = 535)	− 4.42	[− 7.56, − 1.40]	< 0.001
High (*n* = 442)	− 3.67	[− 9.57, 3.94]	0.265
Social support^c^			
Low (*n* = 308)	− 5.43	[− 10.2, − 1.65]	0.010
High (*n* = 683)	− 2.33	[− 6.32, 4.06]	0.461

^a^The risk difference presents the mean change in QALY due to high workload in comparison with normal workload. A risk difference below 0 means more problems with health-related quality of life when having a high workload than having a normal workload

^b^There were *n* = 977 of the individuals included who responded to the control questions

^c^There were *n* = 991 of the included individuals who responded to the social support questions

## Discussion

The workload for home care workers in Sweden is high with more problems reported for quantitative than learning demands in our study. Our study shows that there is a negative effect on HRQoL, with an estimated loss of 3.5% in QALY, for home care workers in Northern Sweden from a high workload compared to a normal workload. This effect can be seen as a moderate negative effect, but nevertheless important to prevent. The results of our study indicate that the personnel whose HRQoL are most affected by a high workload are those experiencing low social support, older personnel with a long tenure, and those who have an education less than assistant nurse. The problems with a high workload affect the health dimensions anxiety/depression and usual activities. It is common with problems of pain/discomfort among home care workers. However, our study does not show that the problems are increased for those who report a higher workload.

In a Swedish study among adults conducted in 2006 in Northern Sweden (Norstrom et al. 2011), a mean QALY score of 0.79 was presented, which is similar to the QALY score of 0.80 among those who report a high workload in our study. Compared to that study, home care personnel with high experienced workload reported more problems with anxiety/depression and less problem for other dimensions of EQ-5D; while for home care personnel with normal workload, less problems were reported for all dimensions. Compared to another study of the Swedish adult population, which was conducted in 2016 (Norström et al. 2019), home care personnel with a normal workload had similar QALY as employed personnel (mean QALY of 0.84). However, problems with anxiety/depression were more common among workers in general and problems with pain/discomfort were more common among home care personnel with normal workload. For home care workers experiencing a high workload, however, the extent of problems with anxiety/depression was also larger than for employed. This potentially larger extent of problems is though likely to be explained by the home care being dominated by female workers as unpublished data from the study by Norström et al. (2019) show similar extent of problems with anxiety/depression for female workers as for home care workers with a high workload. Still, there seems to be differences in how work affects home care personnel in comparison to other workers that are worth noting.

In our study, there was a high proportion (28%) of participants who were defined as having a high workload based on QPSNordic responses. Considering the great extent of pain and discomfort among participants, this indicates that we have at least not underestimated the extent of high workloads within the occupation. In our study, we could identify a moderate effect from a high workload. Previous studies have found a high workload to be associated with several different negative health outcomes when looking at more specific health outcomes, such as stress-related disorders (Nieuwenhuijsen et al. 2010), employee well-being (Bowling et al. 2015) and physical symptoms (Nixon et al. 2011). It is also important to highlight that every third work-related shortcoming in Swedish homecare is estimated to be caused by a high workload, which is twice as high as in the technical field (Arbetsmiljöverket 2015).

### Moderating effect of control and social support

The secondary objective of this study was to test the buffer hypothesis in the demand–control–support model on the relationship between workload and HRQoL. Our results indicate that experiencing high social support reduces the negative health effects on HRQoL due to a high workload, but we did not find any support for the buffering effect of high control. This is in line with a previous study testing the moderating effect on the relationship between job demands and work-to-private-life interference (Viotti and Converso 2016), which also found social support to be to the only job resource to provide a buffering effect. The buffering effect of high social support is estimated to reduce the loss of QALY’s due to a high workload by 3%.

A recent longitudinal study performed in Sweden rejected the buffer hypothesis when testing the buffering effect of social capital and decision latitude on the effect of high psychological demands and burnout at work (Fagerlind Ståhl et al. 2018). The results of our study indicate that high social support is the only resource in the JDC model that has a buffering effect on the relationship between workload and HRQoL.

### Individual factors and workload

Our study indicates that a high workload affects groups of individuals differently. Our results indicate that health care personnel without a relevant health education experience a higher loss of QALY when exposed to a high workload than assistant nurses. With more advanced care being done in old people’s own homes, it could be hypothesised that some of the personnel without a health education lack some important knowledge and skills which makes them more prone to experience the negative effects from a high workload.

Women had a higher risk of problems with both QALY, pain/discomfort and usual activities when compared to men. Our estimates indicated a larger difference in loss of QALY for women than men due to a high workload. Rivera-Torres describes a gender difference where males are only affected by quantitative and not qualitative demands (Rivera-Torres et al. 2013). This is not tested in our study, but could be a potential explanation for this observed difference in QALY loss.

According to Donders, it is important that age is treated as a variable of interest and not a control variable in occupational research (Donders et al. 2012). Although not statistically significant, our results show a tendency to more problems for younger personnel as regards anxiety/depression and usual activities dimensions. This indicates that age could be a protective factor for problems in these dimensions, but the results could also be biased by the “healthy worker effect”, which is defined as the process that allows those who are healthy to remain in certain jobs and forces those with poorer health to leave the job earlier (Hartvigsen et al. 2001).

However, stratified results give a different picture. High age was no longer acting as a protective factor; it was instead associated with a higher loss of QALY and an increased risk of anxiety and depression when exposed to a high workload. Previous research has concluded that age negatively affects the physical work capacity of workers, with a progressive decline in both muscular and aerobic capacity after the age of 30 (de Zwart et al. 1995). This could lead to higher strain for the old health workers, who do not just have to cope with high workload, but at the same time have to deal with declining work capacity. This combination could lead to an increased proneness to the negative health effects related to a high workload. Another occupational-specific explanation to this effect could be that many of those over 64 years old are retired but still work by the hour in home care. Retired staff are often employed by the hour and cover shifts when needed to earn extra money. This means that older workers get to cover sudden changes and sick leaves which force them to primarily work when the work situation in the home care group is already strained.

## Limitations

The first weakness of this study is the cross-sectional study design. According to Häusser, most of the studies supporting the DCS-model have used a cross-sectional design, which also makes it more difficult to imply causality (Häusser et al. 2010). Another issue is the fact that most of the studies that support the DCS model have been cross-sectional, while very few longitudinal studies have found any evidence for the model. This skewed distribution of supportive results makes the choice of study design questionable. Yet, this type of study has not been performed in this setting before, and the aim is not to test for causality, but to describe the current health and workload situation in Swedish homecare. This, combined with the rigorous research that has already been performed in the field, makes the cross-sectional study design sufficient to answer our research questions.

The use of only self-reported data where the participants report both exposure and outcome variables may increase the risk of inflated associations (Theorell and Hasselhorn 2005). To minimise this effect, validated instruments are used to measure both the exposure and outcome variables. Self-reported data are the most common practice in similar studies. According to Theorell and Hasselhorn, objective indicators usually have a weaker association with health when compared to subjective indicators, which implies that our subjective assessments matter (Theorell and Hasselhorn 2005). Furthermore, the association between psychosocial factors and health has been confirmed in multiple studies, which supports the reliability of our results.

Our study is limited to Northern Sweden. There might be problems in applying our results to other parts of Sweden and the world. However, the situation for the personnel is likely to be similar in at least most other municipalities within Sweden. Employees who were on sick leave or absent for other reasons were underrepresented, which would likely present a too low prevalence of high workload in our study. This might bias our findings regarding the relationship between high workload and HRQoL. We expect that, if any, the effect of high workload on HRQoL would for this reason be underestimated. In fact, we expect that including employees who were non-responding and employees who have left the occupation would show a more negative picture on the consequences of high workload within the occupation.

The dichotomization of QPSnordic responses is another limitation that may result in a loss of information. The QPSnordic manual does not clearly indicate how the results should be used or presented, either if all questions are used or their reduced suggestion of questions. The QPSnordic manual claims that both scores from individual items or from scales may be used and that the scores 1–2 and 4–5 possible can be merged together (Dallner et al. 2000). Already, the full version of workload questions will not cover all aspects of workload and that we have further cut down on questions makes it even more limited. This might lead to a too limited aspect of workload, especially as the quantitative and learning demands differ in the extent of reported problems. However, despite this potential risk of valuable information, we trust that our results well should define workload and to be able to represent how personnel’s HRQoL are affected by high workload.

The methodological limitations of this study make it hard to draw any valid conclusions related to workload and health exclusively from this study. However, when combined with the extensive research already done in the field, the study makes an appropriate description of the current situation on workload and health in the home care in Northern Sweden.

### Policy implications and future research

From a managerial perspective, our study shows that the prevalence of both high workload and health problems is high. The potential health benefits of reducing the high workload when it comes to the personnel’s general well-being are estimated to be moderate, but other negative organisational effects due to high workload such as motivation, lower job satisfaction and absenteeism are worth keeping in mind (Sverke et al. 2016).

For those home care groups that are currently facing a high workload, interventions aimed at providing better social support from managers and co-workers may be a valid tool to increase the psychological well-being among home care workers. Due to the high prevalence of high workload, managers are also recommended to reduce the workload as much as possible. Some groups, such as personnel with no relevant health education or those of a higher age, also appear to be more sensitive to a high workload and, therefore, it may be beneficial to focus on these groups when trying to adjust the current workload.

We used QALY in our study, an advantage of which is that we can use our results in health economic evaluations, which enables the results to be used in interventions targeting improved health for home care workers. The results are planned to be used in a future health economic evaluation, where we aim to investigate the cost-effectiveness of increased staffing within the occupation to prevent the health implications of both high workload and high unemployment.

In conclusion, our study shows an association between high workload and HRQoL. The results of our study indicate that the personnel whose HRQoL are most affected by a high workload are those experiencing low social support, older personnel with a long tenure, and those who have an education other than assistant nurse. The home care workers also had a higher prevalence of problems with Pain/Discomfort than previously have been reported for the general population in Sweden.

## Appendix

See Tables 8, 9, 10, 11.

**Table 8:** Responses to workload questions (*n* = 1154)

	Unevenly distributed workload			Too much to do			Too difficult work tasks			Work tasks requiring more education		
	1–2^a^	3^a^	4–5^a^	1–2	3	4–5	1–2	3	4–5	1–2	3	4–5
Gender												
Man	84 (47%)	68 (38%)	28 (16%)	66 (37%)	71 (39%)	43 (24%)	154 (86%)	22 (12%)	3 (1.7%)	107 (60%)	56 (31%)	16 (8.9%)
Woman	329 (35%)	422 (45%)	192 (20%)	266 (28%)	450 (47%)	236 (25%)	865 (91%)	72 (7.6%)	9 (1.0%)	631 (66%)	255 (27%)	64 (6.7%)
Marital status												
Married	285 (37%)	322 (42%)	154 (20%)	228 (30%)	354 (46%)	184 (24%)	695 (91%)	59 (7.7%)	9 (1.1%)	497 (65%)	206 (27%)	59 (7.7%)
Single	126 (35%)	166 (46%)	66 (18%)	104 (29%)	165 (46%)	93 (26%)	321 (89%)	35 (9.7%)	3 (0.8%)	237 (65%)	105 (29%)	21 (5.8%)
Tenure												
< 1 year	49 (51%)	34 (35%)	14 (14%)	44 (45%)	34 (35%)	20 (20%)	84 (88%)	11 (11%)	1 (10%)	55 (56%)	34 (34%)	10 (10%)
1–5 years	131 (40%)	140 (43%)	55 (17%)	108 (33%)	136 (41%)	86 (26%)	293 (89%)	33 (10%)	4 (1.2%)	189 (58%)	98 (30%)	41 (13%)
6–15 years	128 (37%)	144 (42%)	72 (21%)	92 (27%)	169 (49%)	85 (25%)	317 (92%)	26 (7.5%)	2 (0.6%)	230 (67%)	96 (28%)	18 (5.2%)
> 15 years	106 (30%)	171 (48%)	81 (23%)	88 (25%)	181 (50%)	90 (25%)	326 (92%)	24 (6.8%)	5 (1.4%)	265 (74%)	84 (23%)	11 (3.1%)
Health education												
Assistant nurse	280 (36%)	347 (44%)	157 (20%)	220 (28%)	383 (48%)	190 (24%)	726 (92%)	53 (6.7%)	8 (1.0%)	561 (71%)	196 (25%)	34 (4.3%)
Other education	129 (40%)	135 (41%)	59 (18%)	110 (34%)	131 (41%)	82 (25%)	276 (86%)	41 (13%)	4 (1.2%)	163 (51%)	115 (36%)	43 (13%)
Total	416 (37%)	491 (43%)	222 (20%)	334 (29%)	523 (46%)	281 (25%)	1024 (91%)	95 (8.4%)	12 (1.1%)	740 (65%)	314 (28%)	81 (7.1%)

^a^1–2 correspond to the response alternatives “Very seldom” and “Rather seldom”, 3 corresponds to the response alternative “sometimes”, and 4–5 correspond to the response alternatives “Rather often” and “Very often or always”, for the different questions

Responses are presented for all participants, as the questions themselves are not used in separate analyses. Analyses in other tables are limited to those with complete answers. Such limitation is not beneficial for the presentation of the raw data in this table. In Table 1 in the manuscript, the full phrasing of the questions is specified (the first four questions are the same order in that table as in the current table)

**Table 9:** Effect of occupational psychosocial factors and individual characteristics on health-related quality of life (*n* = 1029)

	Crude		Multiple^a^	
	Odds ratio	Confidence interval	Odds ratio	Confidence interval
Mobility^b^				
Work load				
High	1.39	0.80–2.35	1.50	0.86–2.57
Gender				
Woman	1.14	0.58–2.51	1.09	0.54–2.46
Health education				
Other education	1.17	0.67–2.01	1.18	0.64–2.11
Marital status				
Single	0.71	0.38–1.24	0.66	0.35–1.17
Tenure and age				
More than 5 years of experience and ≤ 35 years of age	1.04	0.40–2.39	1.06	0.40–2.50
More than 5 years of experience and 36–54 years of age	0.72	0.35–1.43	0.72	0.33–1.51
More than 5 years of experience and ≥ 55 years of age	1.72	0.92–3.21	1.82	0.94–3.53
Usual activities^b^				
Work load				
High	2.10	1.18–3.68	2.01	1.12–3.55
Gender				
Woman	1.22	0.58–3.01	1.43	0.66–3.60
Health education				
Other education	1.60	0.89–2.83	1.51	0.79–2.83
Marital status				
Single	1.34	0.75–2.37	0.78	0.44–1.43
Tenure and age				
More than 5 years of experience and ≤ 35 years of age	1.61	0.71–3.44	1.86	0.79–4.13
More than 5 years of experience and 36–54 years of age	0.75	0.36–1.52	0.90	0.41–1.93
More than 5 years of experience and ≥ 55 years of age	0.65	0.26–1.43	0.76	0.30–1.75
Pain/discomfort^b^				
Work load				
High	1.06	0.80–1.39	1.10	0.83–1.47
Gender				
Woman	2.25	1.60–3.17	2.05	1.45–2.93
Health education				
Other education	0.94	0.72–1.24	0.86	0.65–1.13
Marital status				
Single	0.77	0.59–1.01	1.16	0.88–1.53
Tenure and age				
More than 5 years of experience and ≤ 35 years of age	0.85	0.56–1.29	0.82	0.53–1.25
More than 5 years of experience and 36–54 years of age	1.89	1.39–2.58	1.82	1.30–2.55
More than 5 years of experience and ≥ 55 years of age	1.90	1.35–2.70	1.84	1.29–2.65
Anxiety/depression^b^				
Work load				
High	2.59	1.94–3.47	2.56	1.91–3.43
Gender				
Woman	1.21	0.83–1.79	1.32	0.89–2.00
Health education				
Other education	1.12	0.83–1.50	1.07	0.77–1.49
Marital status				
Single	1.34	1.01–1.78	1.35	0.998–1.81
Tenure and age				
More than 5 years of experience and ≤ 35 years of age	1.47	0.95–2.28	1.58	0.997–2.50
More than 5 years of experience and 36–54 years of age	1.04	0.75–1.45	1.10	0.76–1.60
More than 5 years of experience and ≥ 55 years of age	0.74	0.50–1.09	0.81	0.54–1.22

^a^Analyses were adjusted for gender, education level, marital status, and tenure and age

^b^Problems were expressed as moderate or severe for each of the EuroQol 5 dimensions. There were 63 who expressed problems with “hygiene”, 52 for “Usual activities”, 602 for “Pain/discomfort”, and 291 for “Anxiety/Depression” for the dimensions

**Table 10:** Effect of high workload on health-related quality of life, as measured with risk difference, depending on workload definition (*n* = 1029)

Health measure	Workload index					
	Median ≥ 3 (*n* = 291)		Mean > 3 (*n* = 105)		Mean > 2.5 (*n* = 293)	
	RD	*p*	RD	*p*	RD	*p*
Quality-adjusted life year scores^a^	− 0.035	0.005	− 0.067	< 0.001	− 0.040	< 0.001
EQ-5D^b^—Mobility^c^	0.025	0.177	0.034	0.281	0.026	0.147
EQ-5D^b^—Usual activities^c^	0.033	0.043	0.049	0.069	0.059	< 0.001
EQ-5D^b^—Pain/discomfort^c^	0.024	0.462	0.050	0.322	0.048	0.142
EQ-5D^b^—Anxiety/depression^c^	0.203	< 0.001	0.296	< 0.001	0.201	< 0.001
EuroQol visual analogue scale^a^	− 5.03	0.007	− 8.00	< 0.001	− 6.00	0.004

*RD* risk difference

^a^A risk difference above 0 means fewer problems with health-related quality of life for those with a high workload than those with a normal workload. *n* refers to the number of individuals defined with a high workload from the definition

^b^EuroQol 5 dimensions. Responses dichotomized to no problems or moderate problems. Problems with each of the dimensions were: 63 for mobility, 52 for usual activities, 602 for pain/discomfort and 291 for anxiety/depression

^c^A risk difference above 0 means more problem with health-related quality of life when having a high workload than having a normal workload

**Table 11:** Diagnostics of the inverse probability weight estimates for the reduced model

	Unweighted^a^			Weighted^b^		
	AbsDiff^c^	SDev^d^	SDiff^e^ (%)	AbsDiff^c^	SDev^d^	SDiff^e^ (%)
Education	0.081	0.458	17.6	0.002	0.451	0.39
Marital status	0.051	0.471	10.9	0.003	0.468	0.61
Gender	0.023	0.370	6.4	< 0.001	0.366	0.02
Age	2.83	13.6	20.7	0.965	13.7	7.0
Tenure						
At most one year	0.019	0.267	7.2	0.032	0.268	11.9
1–5 years	0.081	0.459	17.7	0.032	0.455	6.9
6–15 years	0.021	0.464	4.6	0.002	0.466	0.51
More than 15 years	0.041	0.459	8.9	0.002	0.463	0.41
Tenure and age						
Up to 5 years of experience	0.062	0.486	12.8	< 0.001	0.483	0.10
More than 5 years of experience and ≤ 35 years of age	0.005	0.316	1.6	0.001	0.319	0.38
More than 5 years of experience and 36–54 years of age	0.017	0.460	3.8	0.001	0.459	0.21
More than 5 years of experience and ≥ 55 years of age	0.074	0.397	18.7	0.002	0.410	0.42

^a^Proportions and mean values in unweighted samples are available in Table 2

^b^Estimates after inverse probability weight estimates based on the propensity scores have been applied to balance the groups

^c^The estimated absolute difference (AbsDiff) between those with high workload and those with normal workload for the variable

^d^The standard deviation (SDev) pools those with high workload (“treatment”) and those with normal workload (“control”)

^e^The absolute value of the standardised difference (SDiff) is presented in %
